# Women in chemistry: Q&A with Professor Tricia Breen Carmichael

**DOI:** 10.1038/s42004-024-01287-z

**Published:** 2024-10-01

**Keywords:** Electronic materials

## Abstract

Tricia Breen Carmichael (she/her) is a Professor in the Department of Chemistry and Biochemistry at the University of Windsor.

**Figure:**
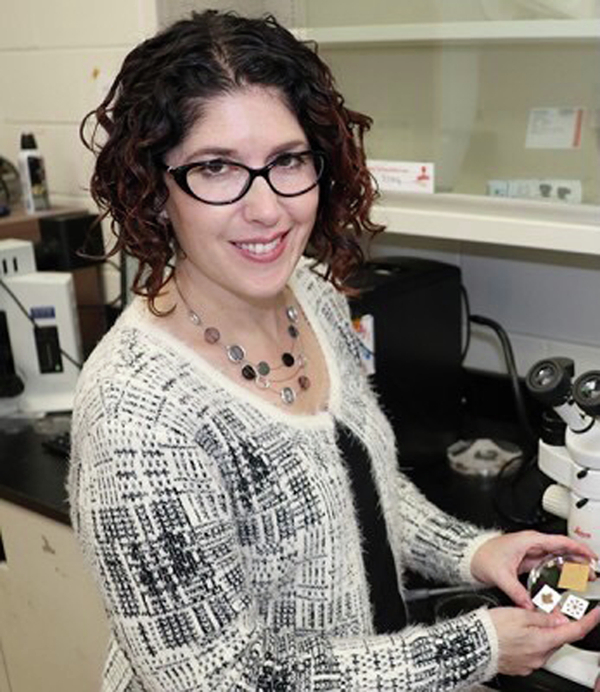
Tricia Breen Carmichael

She received her Ph.D. in 1996 from the University of Windsor, held postdoctoral positions at MIT and Harvard University, and then joined the IBM T.J. Watson Research Center in Yorktown Heights, New York as a Research Staff Member in organic electronics. She currently leads an interdisciplinary research program on stretchable and wearable electronic devices and printed electronics. She has published influential papers in the field, with highlights that include new textile-based wearable electronics (e-textiles), wearable electroluminescent fabrics, soft and stretchable light-emitting devices, and the first transparent butyl rubber for next-generation stretchable electronics. She is the Editor-in-Chief of the journal Flexible and Printed Electronics (Institute of Physics) and the Director of Equity, Diversity, and Inclusion for the Canadian Society for Chemistry.

Tricia has received many awards over her career, and is most proud of those for her EDI work, including the Mary Lou Dietz Equity Leadership Award for demonstrating leadership through contributions to creating an equity culture on campus, the University of Windsor Office of Human Rights, Equity and Accessibility OHREA Award, the University of Windsor Impact Award for co-organizing the first LGBTQ+ in STEM conference in Canada, and the Rotary Peace Chain Link Award.

Why did you choose to be a scientist?

I gravitated toward science in high school. I grew up an only child in a severely dysfunctional family, where gaslighting and invalidation were a part of daily life. When I found science – especially chemistry – in high school, it clicked with me. For me, chemistry had a fundamental truth that no one could dispute. It was a way to understand the world rationally, without the instability and distorted reality that my family used to get through life. When I began my research career as a graduate student, I found that chemistry was so much more than that. My PhD is in synthetic organometallic chemistry and the opportunity to discover new compounds and explore their reactivity was exciting. I then moved on to a postdoctoral position in the lab of George Whitesides, which blew my mind and changed my life. I was initially very intimidated to take on projects that far outside my area of expertise, but gradually, I found that multidisciplinary research in materials science was challenging, stimulating, and fun. Being a scientist has given me an incredible creative outlet, not just with research itself, but also teaching, mentoring, and communication.

What scientific development are you currently most excited about?

It’s hard to choose! Wearable electronics is a rapidly moving field, and the scientific and engineering advances are astonishing. What excites me the most are the translational studies with humans that show how wearables can improve quality of life. I’m fascinated by the work of researchers like John Rogers at Northwestern that bridge the gap between the lab and the person.

What direction do you think your research field should go in?

Wearable electronics brings new and exciting scientific and engineering developments every day, but we must channel that output to address the needs of people in the real world. We need to build collaborations so that the solutions we’re engineering are relevant and useful to people in their daily lives.

I believe that we also need to broaden how we define success in research and develop a culture of acceptance. Science tends to define the most important contributions a researcher can make as publications and citations, and there is a culture of overwork. It causes immense stress to try to live up to an unrealistic and rigid set of expectations, particularly for young researchers. There are so many ways to make meaningful contributions to science and the scientific community – teaching, mentoring, outreach, science communication, to name just a few. We need to value these contributions and support researchers to find their own path. Recognition of the mental health stresses experienced by researchers and non-judgemental support for mental wellness is key.

How would you describe your research philosophy?

I try to approach research with open curiosity. My process is to Google, go down rabbit holes, brainstorm, and bounce ideas off my collaborators. I’m not very strategic when I plan projects, and I don’t try to be. The trap with that way of thinking triggers intense perfectionism, which can make me get stuck in my head. So, an important part of my philosophy is also pragmatic: It’s better to start doing something – running an experiment or writing a paper, for example – even if the experiment might be a terrible idea or the writing might be the worst you’ve ever seen. I give this advice to my students all the time when they get stuck, especially with writing. I tell them to show me their worst thing, because anything is better than being stuck, and it can only get better from there!

What topics do you most enjoy teaching?

I love teaching materials science. At my institution, students take the materials science course in the third year of their undergraduate program. They have a foundation in chemistry, but what makes materials science so fun is that it connects what they have learned to the real world. My favourite materials science topic to teach is semiconductor technologies. My students are astonished when they learn about silicon technologies that they use in their daily lives. I am too, every time I teach this topic! Every year I look forward to teaching materials science and coming up with new ways to evaluate their learning. My favourite the past few years is an assignment called “Express Yourself!”, where I ask the students to choose a material that they find fascinating and use any media method to express what makes it fascinating to them. They’ve surprised and delighted me with podcasts, hand-drawn comic books, commercials, and even songs and poems. Their creativity is marvellous and informative at the same time!

Have you been a minority as a woman at any stage of your career? What was that experience like for you?

I’ve been a minority as a woman in every lab and institution I’ve worked at throughout my career – even now! Early in my career I just thought “I guess that’s how it is”. I persevered, but it was an isolating experience and many times I did think about quitting. It’s not a good feeling. Over the past few years, however, I’ve gotten involved in Equity, Diversity, and Inclusion work to try to improve the culture in chemistry. It has been an unexpectedly rewarding part of my career and life and brought me into an amazing community of people with similar values and goals. I get to see many of them in person only once a year at our national chemistry conference, but even the limited time we have together brings me joy and recharges my batteries. I want everyone in chemistry to have this feeling of belonging.

Where do you hope to see women in chemistry in 20 years?

I hope that we can retire the term “women in chemistry” and just be chemists in chemistry! What I mean is that I hope to see chemistry and the chemical sciences be accessible and welcoming to everyone, regardless of gender, race, ethnicity, sexual orientation, disability, economic status, and other diverse backgrounds. I hope we can work toward a healthy and accepting culture that respects and celebrates people’s differences and values a diversity of contributions. I want our field to grow out of old ideas around work–life balance and prioritize, not stigmatize, mental wellness. By shedding the old ways, we will bring in different perspectives that will only strengthen research and innovation.

*This interview was conducted by the editors of Communications Chemistry*.

